# Prevalence of diabetes mellitus in patients undergoing coronary artery bypass grafting in a Tertiary Care Hospital

**DOI:** 10.12669/pjms.39.4.7250

**Published:** 2023

**Authors:** Bahauddin Khan, Mujeeb Ur Rehman, Mujahid Ul Islam, Imtiaz Ahmad

**Affiliations:** 1 Bahauddin Khan, FCPS Assistant Professor, Department of Cardiothoracic Surgery Rehman Medical Institute, Peshawar, Pakistan; 2 Mujeeb Ur Rehman, MBBS, MS Senior Registrar, Department of Cardiothoracic Surgery, Armed Forces Institute Of Cardiology, National Institute of Heart Diseases (AFIC/NIHD), Rawalpindi, Pakistan; 3 Mujahid Ul Islam, FCPS Associate Professor Rehman Medical Institute, Peshawar, Pakistan; 4 Imtiaz Ahmad, FCPS, Associate Professor Rehman Medical Institute, Peshawar, Pakistan

**Keywords:** Diabetes mellitus, Coronary artery bypass grafting, Gender

## Abstract

**Objective:**

To find out the prevalence of diabetes mellitus in patients undergoing coronary artery bypass grafting (CABG).

**Methods:**

This retrospective study was conducted in Northwest General Hospital and Research center Hayatabad, Peshawar over a duration of 15 months (December 2020 ― March 2022). Every patient was tested through regular laboratory investigations. Diabetes mellitus was established according to WHO standards of fasting plasma glucose >126 mg/dl or two hours postprandial glucose level of 200 mg/dl. Data were analyzed using SPSS. Mean and standard deviation was used for quantitative data. Frequency and percentages were used for qualitative data. Shapiro Wilk’s test was done to find the normality of the data.

**Results:**

Out of a total of 360 candidates, 129 (36%) individuals were non-diabetic and 231 (64%) candidates were diabetic. Among the diabetic patients, 64 (28%) were female and 167 (72%) were males with a ratio of 1:2.6 respectively.

**Conclusion:**

Prevalence of diabetes was 64% among the CABG population. Since the prevalence of diabetes can significantly affect the outcomes of people undergoing CABG, it is essential to generate awareness regarding diabetes among healthcare workers as well as the general population. More extensive research is needed to be carried by various health care centers to figure out the prevalence of diabetes mellitus in cardiac surgery patients.

## INTRODUCTION

In cardiac surgery, diabetic patients represent an important population for the treatment of coronary artery disease. Diabetes mellitus is one of the major risk factors for coronary artery disease. It affects the intraoperative as well as the postoperative course of the patient undergoing CABG. Studies conducted outside Pakistan showed that approximately 30-40% of patients for CABG surgery were found to be diabetics.^1^ This large population of patients has a greater occurrence of illness, death, and prolonged span of hospital stay.^2^,^3^

A systematic review published in 2016-2017 showed that the average prevalence of adult- onset diabetes in our country was 26.3% with prevalence greater amongst males than in females and city zones showing more cases than country regions.^4^-^6^ Individuals having diabetes are more likely to undergo cardiac procedures than those who do not have it. There is a well-established relationship between diabetes and increased morbidity and mortality in patients undergoing cardiovascular operations.^7^-^9^

Diabetic patients undergoing surgical revascularization of coronary atherosclerosis represent a large and complex subgroup of bypass patients. It also presents a challenge to determine the type of bypass procedure they should go through.^7^ Tight glycemic control of diabetes also affects the clinical outcomes so it is necessary to have the data of diabetes prevalence to effectively manage these complicated cases.^1^,^8^ Fewer statistics are available about the prevalence of diabetes in patients who have had a CABG in our country. This research aims to determine the prevalence of diabetes amongst individuals undergoing CABG.

## METHODS

This retrospective research was conducted in the cardiac surgery ward, cardiac operation theatre, and in the cardiac intensive care unit (CICU) of Northwest General Hospital and Research Center, a tertiary care hospital located in Peshawar Pakistan from December 2020 till March 2022, following an endorsement from the hospital research and ethical committee (Ref# NwGH/Res/Ethical Approval/1441, of the institute.

A total of 360 adult candidates scheduled for CABG were included in this research. Every participant was systematically examined through specified laboratory checks. Diabetes mellitus was established according to WHO standards of fasting plasma glucose >126 mg/dl or two hours postprandial glucose level of 200 mg/dl. Standard surgical techniques were used for coronary artery bypass grafting.

Extracorporeal circulation and myocardial protection strategies were implemented as per the attending surgeon. Clinical, angiographic, and procedural data, including complications were documented prospectively through a precisely constructed form and the collected data was analyzed by using the statistical package for social sciences version 25. The incidence and proportion of diabetes were measured and tabulated. The occurrence and ratios were also considered for gender dispersal. All fields were defined in a data dictionary. Post stratification appropriate chi-square test was applied. Two-sided p-value of ≤ 0.05 was taken as criteria of statistical significance.

## RESULTS

**Fig.1:**
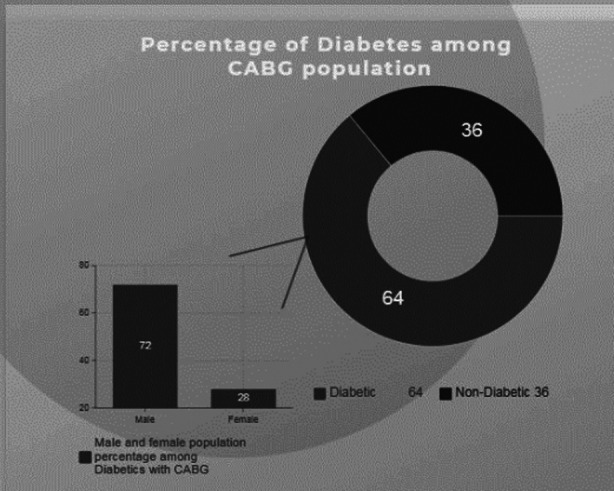
Stratification by Diabetes Mellitus and distribution of gender among the Diabetes Mellitus.

A total of 360 adult patients underwent CABG in the cardiac operation theater of our hospital. Mean age of patients was 57 ± 10 SD (years). There were 276 (77%) male patients and 84 (23%) were female patients in our study. Out of 360, 231(64%) were having diabetes mellitus and 129(36%) were non diabetics patients shown in Table-I. The stratification was again done among the 231(64%) diabetics patients in which 167(72%) were male patients and 64(28%) were female patients with a ratio of 1:2.6 respectively (Fig.1).

**Table-I:** Distribution of Gender, Diabetes Mellitus and stratification of Diabetes Mellitus by gender.

Distribution of Gender n= 360)	Frequency	Percentage	P-Value
Male	276	77%	0.001
Female	84	23%	

*Distribution by Diabetes*	*Frequency*	*Percentage*	

NON DM	129	36%	0.001
DM	231	64%	

*Distribution of gender with respect to Diabetes Mellitus n=231(64%)*	*Frequency*	*Percentage*	

Male	167	72%	0.001
Female	64	28%	

Age, Mean ± SD (years) 57 ± 10.

**Fig.2:**
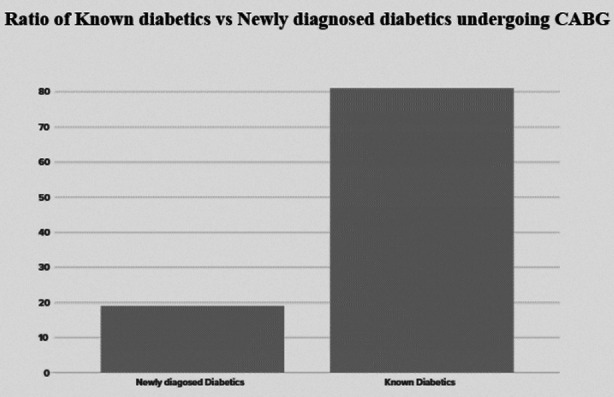
Ratio-of-Known-Diabetics-Vs-Newly Diagnosed-Diabetes-among the male-patients undergoing-CABG.

Out of 167 diabetic male patients, 135 (81%) were known, diabetics. 32 (19%) patients were diagnosed as diabetics on admission (Fig.2).

## DISCUSSION

Diabetes mellitus is one of the major health problems and the prevalence of diabetes mellitus in patients undergoing CABG is variable, subject to cultural or demographic features. In general, most studies in the western countries that include various demographics of patients undergoing CABG surgery showed a range of diabetics from 12% to 30%.^9^-^12^ The incidence of diabetes mellitus in Pakistan is nearly 12%. Discrepancies according to age, gender, position, development, and socio-economic variability are eminent.

Due to a lack of key intermediations, Pakistan is expected to have 11.4 million patients with diabetes mellitus in the next 10 years.^4^,^13^ Cardiac operations in patients with diabetes have been associated with an extended length of hospital stay, higher perioperative illness, and death compared to non-diabetics.^14^ Some postoperative complications are significantly more prevalent among diabetics, mainly renal failure, neurological accidents, sternal dehiscence, and infection.^1^ The main reasons for the predisposition to various infections are due to the strong association between DM and angiopathy, neuropathy, and hyperglycemia.

In addition to increasing the risk for postoperative infections in CABG, DM was associated with a significantly higher incidence of late mortality (up to one year) and related cardiac events.^3^ This is possibly due to the extraordinary occurrence of concurrent diseases like hypertension and renal impairment along with hostile impacts of high blood sugar in cardiac patients.^15^ A study conducted in Spain showed the incidence of diabetes mellitus as 34.9% out of 304 participants undergoing CABG in 24 months which is lower than the incidence found in our study.^1^ Likewise, a study conducted in Brazil showed the prevalence of DM is 36% in coronary artery bypass operation patients with a 70% male population.^3^

Our study also reported the higher prevalence of DM (64%) in patients admitted for CABG with a 72% male population. Another study reported the prevalence of DM (41.8 %) in CABG patients and 59.7% have been reported in male patients which is almost equal to our study.^16^ It is important to note that most research studies demonstrate that the proportion of individuals with diabetes mellitus undergoing CABG operations is increasing.^7^,^9^,^12^ Abramov et al in their research established that the fraction of diabetics had amplified from 18% to 26% in CABG operations performed in the 1990s.^15^

The prevalence of diabetes mellitus in cardiac surgical patients was established to be 64% in our research. These outcomes are different from previously accounted figures reported in the rest of the world.^8^,^12^ The prevalence of diabetics in coronary artery disease has been shown 75.91% and reported 44% in females and 35.6% in males.^17^ Our study reported reported more in in male CABG patients. This high prevalence of patients undergoing CABG with diabetes is alarming. Since diabetes can affect the management as well as outcomes for cardiac surgery patients, it is imperative to have sufficient data to effectively tackle this complex healthcare issue.

Multicenter research work is required to find out the actual prevalence of diabetes in cardiac patients undergoing CABG and other surgical procedures and plan accordingly to benefit the patients. Our study reported the prevalence of newly diagnosed DM was 19% only in male patients. The prevalence of patients with undiagnosed DM was 5.2%. the undiagnosed diabetics are highly valnurable to frequently required resuscitation, reintubation and often showed a longer period of ventilation >one day. Perioperative mortality rate was shown highest.^18^ Another study reported the diabetes diagnosed for the first time in 12% patients.^19^

### Limitation:

A key limitation of this study is that we used a database from a single institution (one center), and although compiled by different surgical teams, this method limits the generalizability of our results.

## CONCLUSION

Our study showed a very high prevalence of diabetes in CABG patients more in male as compared to female patients. Early detection and management of diabetes in patients scheduled for CABG surgery will help to reduce its perioperative and postoperative complications. Multicenter research work and data gathering is required to find out the actual prevalence of diabetes and plan accordingly to improve outcomes for patients undergoing CABG.

### Authors’ Contribution:

**MR and BK:** Designed and conceived with data analysis, manuscript writing and editing.

**MI:** Helped in data collection.

**IA:** Helped in data collection, formal lay out.

**MR:** Proof reading, statistical analysis, and final approval.

**MR:** will be responsible and accountable for the accuracy or integrity of the work.
